# Metastatic Colorectal Cancer

**DOI:** 10.3390/cancers13246346

**Published:** 2021-12-17

**Authors:** Alessandro Passardi, Giorgia Marisi, Paola Ulivi

**Affiliations:** 1 Medical Oncology Unit, IRCCS Istituto Romagnolo per lo Studio dei Tumori (IRST) “Dino Amadori”, 47014 Meldola, Italy; alessandro.passardi@irst.emr.it; 2 Biosciences Laboratory, IRCCS Istituto Romagnolo per lo Studio dei Tumori (IRST) “Dino Amadori”, 47014 Meldola, Italy; paola.ulivi@irst.emr.it

International experts in the study of metastatic colorectal cancer (mCRC) present this series of 14 articles (eleven original articles and three literature reviews).

The treatment options for patients with mCRC have changed considerably in recent years, thanks to the introduction into clinical practice of monoclonal antibodies directed against molecular targets. To date, the following two types of monoclonal antibodies have been approved for clinical use in mCRC: the antiangiogenic agents, Bevacizumab, Ramucirumab and Aflibercept, and the anti-epidermal growth factor receptor (EGFR) antibodies, Cetuximab and Panitumumab. A multicenter Italian observational study (FABIO project) was designed to evaluate the impact of adding the target drugs, Bevacizumab and Cetuximab, to first line chemotherapy in terms of survival and costs. Trial results indicated that first-line biological therapy did not improve long-term overall survival and was associated with higher costs as compared to standard chemotherapy [1]. Gelsomino et al. performed a pooled analysis to evaluate the impact of anti-angiogenics in patients with pre-treated *BRAF*-mutant mCRC. The analysis showed a significant advantage from combining chemotherapy to these agents over the placebo in terms of OS (HR 0.50, 95%CI 0.29–0.85) (*p* = 0.01) [2]. Unfortunately, there are currently no validated markers of sensitivity or resistance to chemotherapy and antiangiogenic drugs. The study by Suenaga et al. showed that single nucleotide polymorphisms (SNPs) in genes involved in nucleotide excision repair (NER) of platinum-induced DNA damage are associated with the superior efficacy of FOLFOXIRI and Bevacizumab, compared with FOLFIRI plus Bevacizumab for mCRC patients in the TRIBE trial [3]. 

Next to antiangiogenic and anti-EGFR agents, the introduction of checkpoint inhibitors provided important achievements in a small group of mCRC patients with microsatellite unstable (MSI-high) tumors. Current results in patients with microsatellite stable (MSS or MSI-low) tumors are disappointing, even if there are several ongoing clinical trials aiming at extending the efficacy of immunotherapy beyond the MSI-high subgroup [4]. In this context, another important aspect is the role of the biological heterogeneity and low inherent immunogenicity of colorectal cancer. The review of Kalanxhi et al. summarized the immunological characteristics of colorectal cancer, the effects that standard-of-care treatments have on the immune system, and the opportunities arising from combining immune checkpoint-blocking therapy with immune-modulating conventional treatments [5].

A topic of interest in the management of mCRC is the evaluation of the response to anticancer treatment. Vera et al. reported the interesting results of the AVAMET study, indicating that computed tomography-based morphological criteria (CTMC) might represent a better marker of pathological response than RECIST in patients with potentially resectable CRC liver metastases [6]. Patients with mCRC rarely develop brain metastases. In these patients, the prediction of survival (< or >6 months) is important for the decision-making process. Rades et al. developed an easy-to-use survival score based on two independent predictors (performance status and absence/presence of non-cerebral metastases), which permitted them to predict survival with higher accuracy than existing tools [7].

Genome-wide DNA methylation of colorectal cancer metastasis revealed a higher methylation status of metastasis with respect to primary tissue, and showed that the methylation pattern of *FIGN, HTRA3, BDNF, HCN4,* and *STAC2* is associated with a poor prognosis [8]. Moreover, high preoperative *KRAS* mutation fractional abundance was demonstrated to be a poor prognostic factor in patients undergoing liver metastasectomy. The combination of preoperative *KRAS* mutation levels with CEA levels seems to give the best prognostic value in this setting of patients [9]. Another study applied a wide integrative omics approach on liver metastasis, and revealed that in synchronous metastasis BRCA1 protein was co-localized in Ito, Kupffer, and endothelial cells in the majority of cases, whereas this occur rarely in metachronous metastases, pointing out a role of BRCA1 as a potential TME biomarker [10].

Furthermore, some studies investigated new potential therapeutic targets in CRC. Iyer et al. suggested that piR-24000 might serve as a potential novel biomarker or a therapeutic target in CRC, specifically in patients presenting with an advanced, aggressive clinical phenotype [11]. Schulte am Esch et al. presented the novel colorectal cancer cell lines BKZ-2 and BKZ-3 as promising cellular in vitro models for colorectal carcinomas and identified the MYC/NMYC molecular pathway involved in cancer stem cells (CSC)-induced carcinogenesis with relevant therapeutic potential [12]. Cancer stem cells (CSC), which are a small subpopulation of tumor cells with high plasticity driving tumor growth and metastasis, are crucial mediators of cancer relapse.

Moreover, another interesting topic is the study of adenoma-carcinoma sequence. Boman et al. investigated the kinetics of adenoma and CRC development in familial adenomatous polyposis (FAP) and sporadic cases in order to identify mechanisms that may explain how changes in tissue dynamics and processes contribute to CRC development. The authors suggests that mutation in the adenomatous polyposis coli (*APC*) gene increases autocatalytic tissue polymerization and induces tumor tissues to autocatalyze their own progressive growth, which drives tumor development in the colon [13].

Finally, there is now the opportunity to integrate genomic and transcriptomic analyses with advanced experimental models. The integration of omics and advanced mouse models of mCRC with more clinical-like criteria of the evaluation of antitumor efficacy in preclinical studies, could accelerate the anticancer discovery process and provide new weapons against cancer [14].
